# Implementation of adolescent HIV screening in two urban pediatric emergency departments in the United States

**DOI:** 10.1371/journal.pone.0321473

**Published:** 2025-04-15

**Authors:** Wei Li A. Koay, Kavitha Ganesan, Justin Unternaher, Sephora Morrison, Shilpa J. Patel, Monika Goyal, Natella Rakhmanina

**Affiliations:** 1 Division of Infectious Diseases, Children’s National Hospital, Washington, DC, United States of America; 2 Department of Pediatrics, The School of Medicine and Health Sciences, George Washington University, Washington, DC, United States of America; 3 ICAP, Mailman School of Public Health, Columbia University, New York, New York, United States of America; 4 Division of Emergency Medicine, Children’s National Hospital, Washington, DC, United States of America; 5 Elizabeth Glaser Pediatric AIDS Foundation, Washington, DC, United States of America; Centers for Disease Control and Prevention, UNITED STATES OF AMERICA

## Abstract

**Objectives:**

Routine, opt-out HIV screening of adolescents and youth (AY) is recommended in the United States in all healthcare settings, including emergency departments (EDs), however, data on ED-based HIV screening among AY remains limited. We aimed to describe the implementation and outcomes of a routine HIV AY screening program in two pediatric EDs in Washington, DC.

**Methods:**

This was a cross-sectional prospective study of an opt-out HIV point-of-care testing (POCT) program of AY aged 13–24 years at a tertiary-based pediatric ED and community-based pediatric ED in Washington DC from March 2009 to February 2019. Descriptive statistics were used to analyze annual program performance by numbers of eligible AY seen, approached, tested, and new HIV identified. One-time ED staff survey collected barriers to HIV screening.

**Results:**

During the 10-year period, out of 191,107 AY seen in ED, 21.9% (n=41,913) were approached for HIV POCT, of which 58.7% were tested (n=24,599); 23 new HIV infections (0.09% of tested AY) were identified. A higher proportion of AY were approached at the community-based ED compared to the tertiary-based ED (58.5% *vs.* 11.4%). The tertiary-based ED experienced a decline in AY approached after shifting the task from designated testers to ED staff. Among 179 surveyed ED staff, the most common barriers to HIV POCT included forgetting to offer the test (41.9%), lack of time (33.0%) and discomfort when approaching parents/guardians (15.6%).

**Conclusions:**

The rate of new HIV diagnoses among screened AY ED patients was <0.1%, however, less than one-quarter of eligible AY were approached for testing. The staff-run HIV POCT model was successful in the lower acuity community-based pediatric ED, while the larger tertiary-based pediatric ED performed well only with the support of dedicated testers. Future studies are necessary to identify the optimal implementation strategy for sustainable ED-based AY HIV screening in the US.

## Introduction

Since 2006, the United States (US) Centers for Disease Control and Prevention (CDC) have recommended that emergency departments (EDs) conduct routine opt-out HIV screening and provide linkage to care for all patients aged 13–64 years regardless of their risk factors [1]. This guidance has been endorsed by the US Preventive Services Task Force (USPSTF) starting at age 15 years and is similarly endorsed by the American Academy of Pediatrics (AAP) [2–4]. Despite these recommendations, HIV screening rates among adolescents and youth (AY) in the US are low, with only 6% of high school students ever having been tested for HIV [5] and 22–23% of high school students who ever had sexual intercourse and adolescent men who have sex with men had ever been tested for HIV [6,7].

Pediatric EDs play a vital role in addressing the ongoing HIV epidemic among AY who tend to access EDs for medical care [8,9]. AY aged 13–24 years continue to account for almost 20% of new HIV infections in the US [10], and over half of AY with HIV remain unaware of their diagnosis [11]. This highlights the ongoing need for increased HIV testing among AY which can be facilitated through leveraging the capacity of pediatric EDs to offer HIV screening through point-of-care testing (POCT). Routine HIV screening in ED settings can help to identify HIV infection among AY without a known HIV exposure or symptoms of acute HIV infection [12,13], also providing an opportunity to initiate rapid antiretroviral therapy in AY with newly diagnosed HIV [14]. Various models have been identified to optimize and integrate routine HIV screening into ED settings, however the majority of these models have focused on adults [15–17]. Studies that target routine ED HIV screening in pediatric healthcare settings have shown that acceptance rates are high and that screening in urban pediatric EDs can be successfully implemented [13,18–20]. However, sustainable and effective implementation of HIV screening in pediatric ED has not been reported to date and requires a multisystem approach to be successful.

Access to HIV screening is highly relevant in Washington, District of Columbia (DC), one of the “hot spots” of the US HIV epidemic [21], where 1.7% of residents live with HIV as of 2021 [22] and 1 in 7 new HIV diagnoses are among AY [23]. The 2021 Youth Risk Behavioral Survey showed that 28.5% (*vs.* 30.0% nationwide) of DC high school students have ever had sexual intercourse, 18.0% (*vs.* 20.7% nationwide) were currently sexually active, and 6.2% (*vs.* 6.0% nationwide) have had sexual intercourse with ≥4 partners during their lifetime [24]. Having high engagement in sexual activity with multiple partners predisposes AY to having a higher risk of HIV acquisition [25]. We describe the implementation process and challenges of ED-based POCT for HIV and report on the implementation outcomes of routine HIV opt-out screening of AY in two very diverse urban pediatric EDs spanning a 10-year period in an area of high HIV prevalence.

## Methods

### Study design and setting

This prospective cross-sectional study of the implementation of HIV screening of AY (aged 13–24 years) through ED-based POCT was conducted in a tertiary-based and a community-based pediatric ED located in Washington, DC. We analyzed the annual performance of the HIV screening program from its launch date of March 1^st^, 2009 through February 28^th^, 2019, and conducted a staff survey to identify barriers to HIV screening. From herein, each “Year” described includes 12 months of data from March of the year of interest to February of the subsequent year (e.g., Year 1 = March 2009 to February 2010, etc.).

The tertiary-based ED (TBED) is located within a free-standing, quaternary care pediatric hospital and the community-based ED (CBED) is located in a part of the city with high HIV prevalence with lower acuity patients and volumes that are approximately one-third that of TBED [22]. Combined, both EDs accommodate approximately 120,000 visits annually, of which ~15.8% consist of AY (13–24 years of age) who are predominantly of racially and ethnically minoritized populations (>80%). This study was approved by Children’s National Hospital’s Institutional Review Board (Pro00000274) which included the use of information sheets where individuals could verbally decline data collection of their standard of care HIV screening in the ED, and where ED staff could verbally consent to complete a quality improvement survey.

### Program implementation

Routine opt-out HIV POCT using the OraQuick Advance Rapid HIV-1/2 Antibody Test kits (OraSure Technologies, Inc., Bethlehem, PA) was implemented at TBED in March 2009 and at CBED in October 2010. The OraQuick HIV POCT is a non-invasive test which uses oral fluid collected with a swab and results within 20 minutes. Test kits were initially provided at no cost by the DC Department of Health (DOH) until the program transitioned to a billable clinical service in 2015. In the event of a positive HIV POCT result, a confirmatory Western Blot (before 2014), 4^th^ generation HIV antigen/antibody test (from 2014) or HIV nucleic acid test (NAT) from blood was conducted.

### ED HIV POCT algorithm

**Fig 1:**
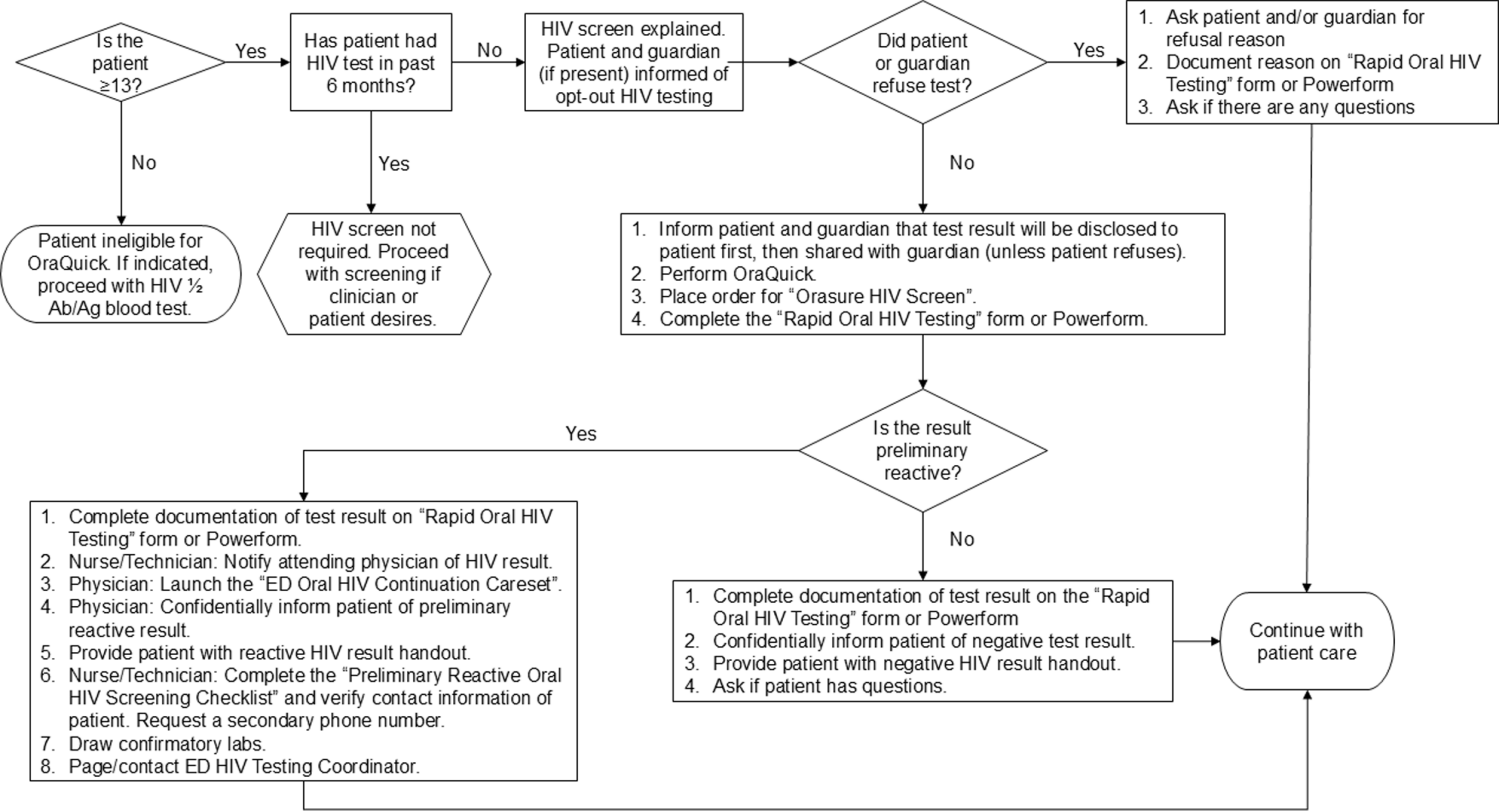
HIV screening algorithm.

TBED and CBED utilized identical HIV screening algorithms and Cerner electronic medical records (EMR) for clinical care (Fig 1). In DC, written consent is not required for HIV testing of adolescents [26]. Test results were reported to the adolescent regardless of age and were shared with their guardian only after obtaining the verbal consent from the adolescent. The HIV POCT algorithm was designed to fit a pediatric setting based on the results of a pre-implementation survey of AY seen in the ED and their accompanying guardians [27,28]. To maximize acceptance of HIV screening in this population, the algorithm incorporated informing the minor adolescent (<18 years of age) and accompanying guardian that the test results would be shared only after adolescent’s verbal consent (Fig 1).

ED staff members approached all AY ≥13 years of age, and their accompanying guardian if present, in triage or in the ED room by informing them that an HIV rapid oral fluid test would be performed on them unless they declined. HIV screening was deferred if the patient reported having a known HIV infection or having taken an HIV test in the past six months without any known risk factors for HIV acquisition since the last test. TBED and CBED use the Emergency Severity Index (ESI) to triage patients, with 1 being the highest resource level, requiring immediate life-saving attention, and 5 indicating non-urgent and not requiring resources. Patients triaged as ESI 1 and patients with altered mental status or acute psychiatric conditions were not approached for HIV screening. The AY was considered an “opt-out” if they declined HIV screening and/or if the guardian (when present with minor adolescents) declined the screening. The ED staff documented the reasons that AY or guardians declined an HIV screen and the reasons for why AY were not initially approached for screening. If POCT was declined by the accompanying guardian, an information sheet was provided to the AY describing how to access confidential free of charge HIV testing in the metropolitan DC area.

ED HIV POCT was performed after verbal consent and the results were sent to hospital’s central laboratory and entered in the patient’s EMR. An electronic order entry (Powerform) was introduced via the Cerner EMR system in June 2016 (Year 8). The Powerform automated the HIV screening process by electronically attaching a sequence of action steps (including consent, test results, confirmatory testing for those with reactive HIV results, and referral pathway) for all eligible patients registered in the ED. In the case of a negative HIV POCT result, AY were provided with brief post-test counseling including written information on HIV and sexually transmitted infections (STI) risk reduction. AY who had a positive HIV POCT result received confirmatory testing for HIV, individual counseling with an attending ED physician and case manager and were linked to specialized HIV services located at the hospital’s main campus within 48–72 hours. ED HIV program staff were notified of every positive HIV POCT result for follow up and name-based reporting of new confirmed HIV cases to the DC DOH.

### Staff survey regarding ED HIV POCT

An interim analysis in August 2011 for ED HIV POCT reported a low rate of HIV screening of AY, with an average of only 5% of eligible patients approached for screening (programmatic data, unpublished). As part of quality improvement and evaluation of the ED HIV screening program to identify reasons for low rates of HIV screening, an online survey with 25 multiple choice and Likert Scale questions assessing knowledge of HIV and barriers to HIV screening was administered to TBED and CBED staff (physician, nurses, technical staff) from June 1^st^, 2013 to October 31^st^, 2013.

### Data analysis

HIV screening data from both EDs were collected for programmatic needs and for this research study. We collected reasons for not approaching AY for HIV POCT, primary reasons from AY for declining screening, HIV POCT results and confirmatory HIV PCR results. Data were collected via paper forms (before 2016) and the electronic Powerform (from 2016) and recorded into a de-identified database to quantify approach and screening rates. Confirmed HIV infection as well as HIV positivity rates (i.e., proportion of tests conducted that were confirmed to be HIV infections) were calculated. Demographics (age, gender, race, ethnicity) were collected on all AY who received HIV screening. Patient volumes for adolescents eligible for screening and their chief complaints for ED visit were obtained from the ED electronic database and EMR, respectively. Chief complaints for confirmed HIV infections were categorized as symptomatic or asymptomatic for acute HIV infection based on report of flu-like symptoms [29]. Descriptive statistics were used to analyze the HIV screening and staff survey results.

## Results

### Study participant characteristics

From March 2009 through February 2019, 191,107 AY were eligible for HIV screening at both EDs. Of those eligible, 21.9% (n=41,913, median age 16.2 years (interquartile range (IQR) 14.7, 17.6), 90.3% Black, 39.2% male) were approached for routine HIV screening. Over half of those approached received an ED HIV POCT (n=24,599, 58.7%), of whom 0.13% (n=50) had a positive HIV POCT result. Of the AY with a positive HIV POCT, 0.09% (n=23) were confirmed to have HIV, all of whom were promptly linked to HIV care. TBED had a high volume of eligible AY (n=148,324), but only approached 11.4% of their patients (n=16,869). CBED had 42,783 eligible AY, of whom over half were approached for HIV POCT (58.5%, n=25,044) (Table 1).

**Table 1:** Outcomes of the HIV screening program at two urban pediatric EDs in Washington, DC.

Program Performance March 2009-February 2019^*^	CBED^*^	TBED	Total
**Total adolescent patients seen in ED**	42,783	148,324	191,107
**Total patients approached (% of seen in ED)**	25,044 (58.5)	16,869 (11.4)	41,913 (21.9)
**Median age, years (Q1, Q3)**	16.0 (14.6, 17.2)	16.5 (14.9, 18.0)	16.2 (14.7, 17.6)
**Black, n (%)**	24,644 (98.4)	13,172 (78.1)	37,836 (90.3)
**Male, n (%)**	9,893 (39.5)	6,516 (38.6)	16,409 (39.2)
**Total patients tested (% of approached)**	12,422 (49.6)	12,177 (72.2)	24,599 (58.7)
**Reactive HIV screening results (% of tested)**	17 (0.20)	33 (0.27)	50 (0.13)
**Confirmed new HIV positives (% of tested)**	10 (0.08)	13 (0.11)	23 (0.09)
**Median age, years (Q1, Q3)**	17.6 (16.3, 17.9)	16.8 (16.6, 18.6)	17.5 (16.6, 17.9)
**Black, n (%)**	10 (100.0)	12 (92.3)	22 (95.7)
**Male, n (%)**	7 (70.0)	7 (53.8)	14 (60.9)

* HIV screening initiated at CBED in October 2010.

Reasons for not approaching eligible AY were documented for a limited number (n=3,896) of patients, with the most common reasons including staff lacking time (23.8%, n=927), report of recent completion of an HIV test (23.2%, n=902), patient’s medical status (19.5%, n=759) and patient’s inability to consent due to medical or mental status (19.3%, n=753). TBED tested 72.2% (n=12,177) of approached AY, while CBED tested almost half (49.6%, n=12,422) of approached AY (Table 1). The most common reasons for AY and/or guardian declining HIV POCT included having a recent negative HIV test (30.3%, n=4,838) and not thinking they were at risk for HIV (27.9%, n=4,459).

Thirty-three AY (0.27% of tested) had a positive HIV POCT at TBED, of whom 13 (0.11%, median age 16.8 years (IQR 16.6, 18.6), 92.3% Black, 53.8% male) were confirmed to have HIV. At CBED, 17 AY (0.20% of tested) had positive HIV POCT results, of whom 10 (0.08%, median age 17.6 years (IQR 16.3, 17.9), 100% Black, 70% male) were confirmed to have HIV. Less than half (46.0%) of all positive HIV POCT results from both EDs were confirmed to be true positives with a positivity rate of confirmed HIV infections over the 10-year period being 0.09% (n=23). The median age of all confirmed new HIV positives was 17.5 years (IQR 16.6, 17.9), the majority were Black (95.7%, n=22) and over half were male (60.9%, n=14) (Table 1).

**Fig 2:**
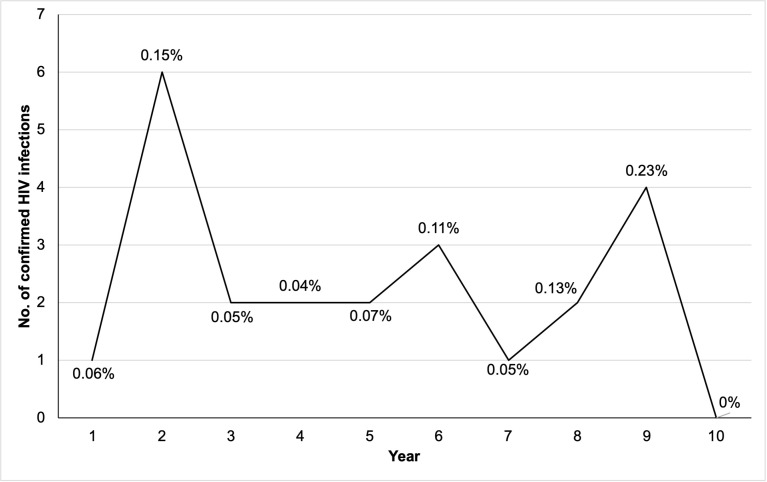
Number of confirmed HIV infections and positivity rates identified through the screening program at both EDs by year.

The majority of AY who were newly diagnosed with HIV presented to the ED with a chief complaint of symptoms associated with acute HIV infection (69.7%; n=16), with fevers/chills/night sweats (39.1%, n=9) being the most common symptom at the time of presentation. Seven AY with newly diagnosed HIV would have been missed if targeted HIV screening based on clinical symptoms was offered (Table 2). Years 2 (2010–2011) and 9 (2017–2018) identified the highest number of new HIV infections over the 10-year period, with annual positivity rates ranging from 0–0.15% during the study period (Fig 2).

**Table 2:** Presenting symptoms of adolescents with confirmed HIV infection (n=23).

	n (%)
**Flu-like or STI symptoms (n=16)**	
Fevers/chills/night sweats	9 (39.1)
Headache	3 (13.0)
Rash	2 (8.7)
Nausea/vomiting/diarrhea	4 (17.4)
Myalgia	2 (8.7)
Sore throat	3 (13.0)
Fatigue	5 (21.7)
Lymphadenopathy	6 (26.1)
Vaginal/penile discharge or dysuria	3 (13.0)
**Non-flu-like symptoms (n=7)**	
Trauma	2 (8.7)
Cellulitis	2 (8.7)
Constipation/abdominal pain	2 (8.7)
Asymptomatic	1 (4.3)

### HIV POCT implementation and outcomes at TBED

**Fig 3:**
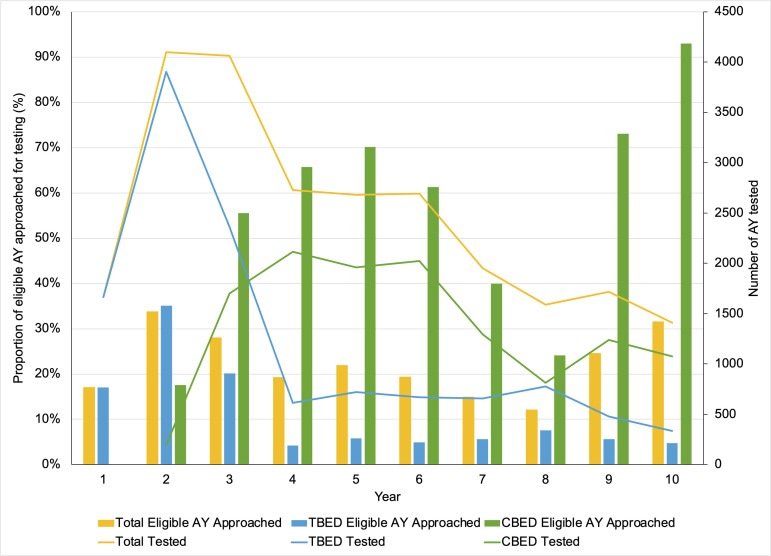
Proportion of eligible adolescent and youth (AY) approached for HIV screening and number of AY who were tested for HIV by ED site (Mar 2009 – Feb 2019).

At TBED, the ED HIV POCT program was originally implemented using a physician-generated order in March 2009 with ED staff conducting HIV POCT. During the first three months of implementation, only 169 AY received HIV POCT. This low HIV screening rate prompted the rapid engagement of two Young Adult Health Advocates (designated testers) in the ED who were funded through a Ryan White Early Intervention Services (EIS) award in June 2009. The impact of the designated testers was quickly visible with a significant rise in the number of approached and screened AY within their first month. In 2010, the task of ordering HIV POCT was transitioned to the ED nurses to facilitate workflow. Designated testers, however, continued to proactively identify eligible patients and worked in synergy with nursing staff to administer the POCT and deliver the test results. The high performance of HIV POCT was sustained during Years 1–3 (2009–2012) with 17–35% of eligible AY approached for HIV screening (Fig 3). By September 2011 (Year 3), following a series of staff training, the task of HIV POCT became entirely staff-run by ED nurses, physicians and technicians, resulting in a decline to only ~5% of eligible AY being approached for HIV POCT. Low HIV screening rates were observed from Year 4 (2012–2013) onwards and was not affected by HIV POCT trainings, staff sensitization trainings and HIV screening campaigns. There was a slight increase in HIV screening rates during Year 8 (2016–2017) to 7.5% after the electronic medical record HIV POCT orders within the Powerform was introduced in 2016 and HIV screening champions were identified among ED staff, however, this increase was not sustained in subsequent years (2018–2019).

### HIV POCT implementation and outcomes at CBED

In October 2010, HIV POCT was implemented at CBED as a staff-run model without the support of designated testers and to date continues to be maintained by ED nurses, physicians and technicians. The HIV POCT program at CBED maintained high levels of performance, consistently approaching >50% of eligible AY since its launch. During Years 3–6 (2011–2015), 50–70% of eligible AY were approached. Surprisingly, following the implementation of the electronic medical record Powerform in June 2016 (Year 8) to place HIV POCT orders, the proportion of eligible AY who were approached for screening dropped down to 24.1% during that year. To address this decline in HIV screening, ED HIV screening champions (nurses and physicians) were identified later in 2016 (Year 8), in addition to additional staff trainings to increase HIV screening rates. As a result, during Years 9–10 (2017–2019) there was a significant increase in the proportion of eligible AY being approached for HIV screening at CBED to 93% (Fig 3).

Ongoing training of new staff to conduct HIV POCT, regular retraining of already certified ED staff, regular meetings with ED staff and leadership to promote screening and frequent HIV screening campaigns took place at both EDs throughout the study period.

### Staff-reported barriers to HIV POCT

**Fig 4:**
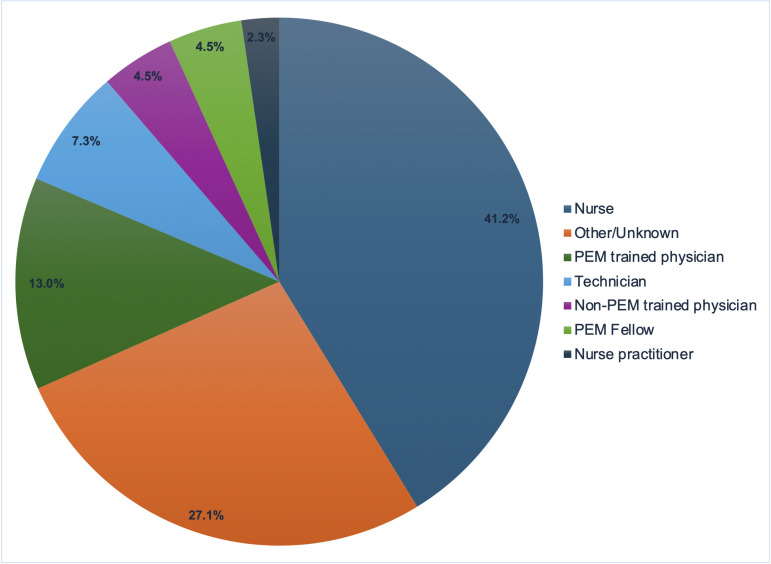
ED staff roles among survey respondents for barriers to screening. Acronyms: PEM = Pediatric Emergency Medicine.

In Year 5 (2013–2014), a total of 179 TBED and CBED staff (38.4% aged 31–40 years, 16.6% male, 53.5% White, 25% Black) completed an online survey assessing barriers and challenges to ED HIV screening. Of the 145 people who reported on their work location, the majority (55.9%, n=81) worked only at TBED, one-quarter worked at both TBED and CBED (24.8%, n=36), and one-fifth worked only at CBED (19.3%, n=28). Most respondents were nurses (40.8%; n=73) followed by pediatric ED attending physicians (12.8%; n=23) and technicians (7.3%, n=13) (Fig 4).

**Fig 5:**
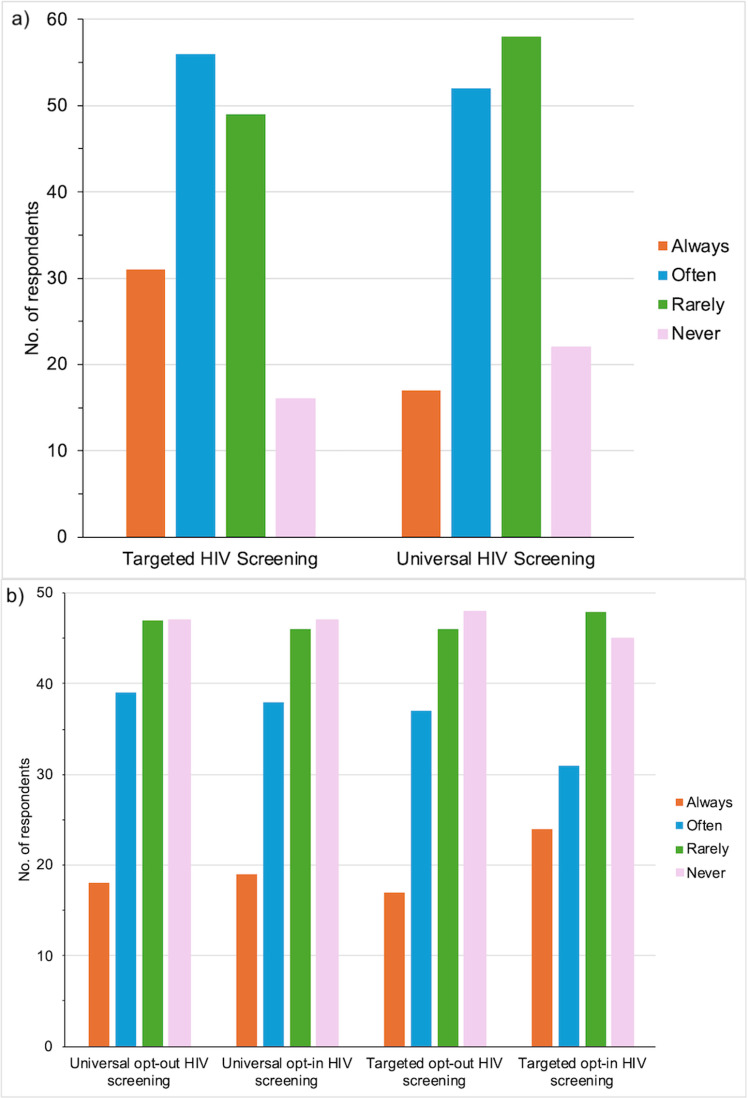
HIV screening approaches undertaken by Pediatric ED Staff a) in general (n=153) and b) in the month prior to being survey (n=151).

Approximately three-quarters of respondents (76%; n=136) were aware of the CDC recommendations for routine HIV screening in the US, but only 36.9% (n=66) were aware that the hospital had a policy on routine opt-out HIV screening for AY ≥13 years of age. In general, most respondents reported always (20.3% *vs.* 10.8%) or often (36.6% *vs*. 31.9%) performing targeted HIV POCT based on their risk assessment of the patient as opposed to routine HIV screening with POCT (Fig 5a). Interestingly, during the month preceding the survey, the majority of respondents reported rarely or never offering any form of HIV screening to AY (Fig 5b). The main barriers to HIV POCT reported were forgetting to offer the test (42%; n=75) followed by lack of time and competing priorities with other ED tasks (33%; n=59), and discomfort with approaching accompanying guardians of AY (15.6%, n=28) (Table 3). When asked for suggestions to increase the number of AY tested for HIV, an overwhelming number of respondents requested reminders to offer and order HIV POCT for AY *(“lots of reminders”, “keep us reminded”, “to increase the number I screen I mostly just need to remember”*). Common ***systems-based themes*** that emerged included addressing staffing responsibilities and education, incorporating screening into the regular ED workflow and implementing automated electronic prompts to remind staff. A selection of relevant comments included *“repeat refresher on guidelines”,* “*incorporate it into ‘normal habits’ within scope of patient care*”, *“have testers in the ED”* and *“automatic orders in Cerner”*. ***Patient-related themes*** included having signage in the ED about HIV screening *(“post info for patients in waiting area”, “put signs in rooms prompting patients to ask for the test”*) and normalizing HIV screening by offering it to all patients *(“normalize it as something we do for all adolescents”,* “*make uniform guidelines for opt-out screening so that psychosocial barriers are not present with parents”*). Feedback from this survey on the need for automated electronic prompts resulted in the development of the Powerform order set in 2016.

**Table 3:** Staff-reported barriers to HIV screening (n=179).

Barrier	Likert Responses (n=179)	
	Always/Often n (%)	Rarely/Never n (%)
Test kits are easy to access	121 (67.6)	10 (5.6)
Challenges with Cerner orders	16 (8.9)	113 (63.1)
Difficult in accessing testing supplies	6 (3.3)	120 (67.0)
Lack of training in how to conduct test	16 (8.9)	111 (62.0)
Lack of training on providing negative test results	11(6.2)	118 (65.9)
Lack of training on providing reactive positive test results	22 (12.3)	109 (60.9)
Legal burden of dealing with minors	18 (10.1)	112 (62.6)
Concerns about linking to follow up care	18 (10.1)	109(60.9)
Lack of time/competing priorities	59 (33.0)	69 (38.6)
Forgetting to offer the test	75 (41.9)	56 (31.3)
Uncomfortable approaching adolescents	15 (8.4)	117 (65.4)
Uncomfortable approaching parents/guardians	28 (15.6)	102 (57.0)
Other	6 (3.4)	41 (22.9)

Targeted HIV screening refers to approaching a patient based on being high-risk for HIV on the basis of behavioral, clinical or demographics. Routine HIV screening refers to performing HIV tests for all patients regardless of their risk level for HIV acquisition. Opt-out screening is the conduct of HIV test unless a patient declines. Opt-in screening is offered to the patient and allowing the patient to decide if they would like to be tested. Non-respondents included those who skipped the question and administrative staff.

## Discussion

We report on a pediatric ED HIV screening program in two diverse urban areas in a city with high HIV prevalence where we were able to provide HIV POCT and sexual health counseling to over 40,000 AY and their guardians during the 10-year study period. Despite missed opportunities to provide counseling and HIV screening for all eligible AY, the implementation of this routine ED HIV POCT program has led to the expansion of sexual health services in the hospital, opportunities to provide education regarding HIV/STI prevention including HIV pre-exposure prophylaxis (PrEP) [30] and the introduction of the still ongoing condom distribution in both TBED and CBED through the support of the DC DOH.

Our pediatric ED HIV POCT program diagnosed HIV in 23 AY, several of whom were asymptomatic for acute HIV and would have otherwise gone undiagnosed. Based on our HIV incidence and HIV screening numbers, we estimate that >75 AY with HIV might have missed opportunities for identifying and treating their infection. Timely HIV diagnosis and linkage to care has been shown to improve short- and long-term HIV treatment outcomes [31,32]. The identification of HIV in these AY not only allowed us to optimize their health outcomes, but also served as a means to decrease HIV transmission in DC, where AY aged 13–24 years with HIV comprise 0.3% of the population [22]. Between 2016 and 2020, AY represented over 19% of new HIV cases in DC, with the proportion of new HIV diagnoses among young adults aged 20–24 years remaining stagnant during these five years [22]. The low incidence of new HIV diagnoses (0.09%) among tested AY during the 10-year period in our study was lower than we had anticipated and in comparison with a pediatric ED from Atlanta, Georgia (0.43%) which is also an area of high HIV prevalence and an urban pediatric ED in Cincinnati, Ohio (0.25%) [33,34]. It should be noted that we did not perform further HIV testing on the AY with negative OraQuick test, and that some cases of acute HIV infection could have been missed by using POCT screening unless provider selected HIV PCR test for targeted testing. While our lower rate of HIV detection could be related to the sustained decline in HIV screening at the TBED since 2011, it is most likely related to the overall decline in number of new HIV infections in DC including among AY, largely due to regional widespread HIV screening and educational and awareness campaigns led by DC DOH and multiple regional stakeholders.

Our data indicated a high false positive rate for HIV POCT (3.4 per 5,000) despite a reported false positive rate of 1 in 5,000 for OraQuick [35]. Our high false positive rate might be indicative of user-induced error or self-reported patient food/drink behavior, despite multiple trainings and recertifications among ED staff [35]. Specificity rates can also decrease as test kits approach expiration which can be more prominent with infrequent usage [36]. This challenge can be rectified by better forecasting of POCT test kit usage, improving quality controls and frequent refresher trainings for ED technicians performing the test.

Published studies, including our data, suggest high acceptance of ED-based routine HIV screening by adults and adolescents [37–39]. However, the majority of EDs, especially pediatric EDs, do not provide HIV screening in a sustainable manner [16,40,41]. In our experience of implementing routine HIV POCT screening among AY since 2009, we encountered significant differences in the performance of a similarly modeled HIV POCT program in two different ED settings. Consistent with adult data from community healthcare settings [42], the ED staff-run model maintained steady performance at the lower acuity smaller pediatric ED based at the community hospital. The larger, higher acuity and higher volume pediatric ED HIV POCT screening performed very well in the presence of the designated testers, however, was unable to maintain the level of performance with ED staff support only. The staff in the larger and busier ED reported forgetting HIV screening and seeing it as a competing priority with other clinical duties. Several adult studies have also shown higher efficiency of HIV screening in healthcare settings and linkage to care across urban and rural settings in the US when using dedicated testers (counselor) *vs.* provider-based screening [43–45].

To respond to the challenges of the performance of the ED HIV POCT screening at TBED, we designed an automated HIV POCT screening icon reminder in the EMR for every patient ≥13 years of age registered in our EDs. Implementation of the electronic HIV screening form in Year 8 (2016) led to an increase in the proportion of eligible AY being approached for HIV POCT at both EDs, however, this increase was sustained only at the CBED. The lack of sustained improvement in HIV screening rates at TBED despite the EMR reminder, Powerform and staff training activities may be attributable to their higher patient acuity and volume, suggesting an ongoing for designated testers.

Nationwide, less than 25% of pediatric ED physicians and staff report that they have rapid HIV testing available in their EDs [37,46]. To effectively implement routine HIV screening as a component of the healthcare continuum for adolescents, it will require the availability of expedited HIV testing and increased level of comfort among pediatric ED providers in addressing HIV screening and overall sexual and reproductive health issues [37,40,47]. Except for specialized adolescent providers, pediatric providers tend to be reluctant to expand their services to include HIV and STI screening in adolescents and address the issues of reproductive health on a regular basis [39,46]. Even after 4 years of routine HIV POCT screening in our EDs, we were surprised to find through our survey that targeted HIV testing *vs.* routine HIV screening of AY was still favored by the majority of the pediatric ED staff. The preference of the pediatric ED staff towards targeted screening is particularly concerning due to the lack of specific clinical symptoms for acute HIV infection in many people with HIV who present to the ED and a limited capacity of medical personnel to fully evaluate history of risk behaviors in busy ED settings. This is consistent with provider feedback from pediatric and adult EDs in New York City where HIV screening was highly acceptable by adolescents, however, providers were sometimes unwilling to offer HIV screening and may discourage youth from HIV screening if they believed them to be at low risk [48]. Unfortunately, due to several logistical issues, we have not repeated staff surveys which could provide insights into the evolution of facilitators and barriers of ED HIV POCT. More education and training are needed to combat stigma towards HIV testing and the importance of HIV screening even without clinical symptoms.

To ensure sustainability of our ED HIV POCT program, in Year 6 (2015–2016) we made an important transition from grant support to third party reimbursement and simultaneously expanded our opt-out HIV POCT program to include routine STI screening. We began billing for HIV POCT after assessing pertinent billing practices, reimbursement models, and programmatic information with a multidisciplinary task force (i.e., ED and ID faculty, laboratory medicine, hospital administration). Transition to a non-grant supported model required us to fully rely on the ED staff to support the HIV POCT program, which was successful in the community-based ED but not sustainable at the larger ED (TBED). Our ED HIV POCT program has continued to evolve in recent years and the hospital has continued to develop an even more robust sexual and reproductive health network for the AY living in the metropolitan DC area. In collaboration with the Division of Adolescent and Young Adult Health and HIV Prevention and Treatment Services, a weekly Sexual Health clinic was launched to provide STI management and PrEP counseling for AY seen at ED with negative HIV or positive STI tests. Additionally, outreach coordinators are dispatched to TBED weekly to support HIV/STI education efforts and referrals for AY to the hospital’s Sexual Health Clinic.

## Conclusions

Routine opt-out HIV screening of AY in pediatric CBEDs was feasible to implement and sustain in areas of high HIV prevalence. During our 10-year experience, the staff-run HIV POCT model proved successful in the lower acuity CBED, while the larger and busier TBED performed well only with the support of dedicated testers. The incidence of HIV among screened AY ED patients was low, however, less than a quarter of eligible AY were approached.

The success of implementing routine opt-out AY HIV screening in pediatric EDs must consider the availability of time and human resources, staff education and buy-in, and needs to include detailed pre-implementation planning and resource allocation. While not addressed in this study, HIV and STI awareness among AY and their guardians also has the potential to improve uptake of routine HIV screening [49,50]. In the US and globally, a significant proportion of new HIV infections occur in populations not identified as high risk and are hence not targeted for prevention approaches including HIV screening, which further justifies the need to routine HIV screening among AY [51].

Addition of HIV screening into the spectrum of preventive activities within pediatric EDs has the potential to expand access to sexual and reproductive health counseling and STI and HIV testing among AY in the US. While undoubtedly carrying a benefit of early identification of HIV infection, routine HIV screening in pediatric EDs also provides an excellent opportunity to increase awareness of and access to HIV and STI screening, treatment and prevention among AY.
